# Bioactivity of the *Murex* Homeopathic Remedy and of Extracts from an Australian Muricid Mollusc against Human Cancer Cells

**DOI:** 10.1093/ecam/nep042

**Published:** 2010-10-20

**Authors:** Kirsten Benkendorff, Cassandra M. McIver, Catherine A. Abbott

**Affiliations:** School of Biological Sciences, Flinders University, GPO Box 2100, Adelaide, 5001, Australia

## Abstract

Marine molluscs from the family Muricidae are the source of a homeopathic remedy *Murex*, which is used to treat a range of conditions, including cancer. The aim of this study was to evaluate the *in vitro* bioactivity of egg mass extracts of the Australian muricid *Dicathais orbita*, in comparison to the *Murex* remedy, against human carcinoma and lymphoma cells. Liquid chromatography coupled with mass spectrometry (LC-MS) was used to characterize the chemical composition of the extracts and homeopathic remedy, focusing on biologically active brominated indoles. The MTS (tetrazolium salt) colorimetric assay was used to determine effects on cell viability, while necrosis and apoptosis induction were investigated using flow cytometry (propidium iodide and Annexin-V staining, resp.). Cells were treated with varying concentrations (1–0.01 mg/mL) of crude and semi-purified extracts or preparations (dilute 1 M and concentrated 4 mg/mL) from the *Murex* remedy (4 h). The *Murex* remedy showed little biological activity against the majority of cell lines tested. In contrast, the *D. orbita* egg extracts significantly decreased cell viability in the majority of carcinoma cell lines. Flow cytometry revealed these extracts induce necrosis in HT29 colorectal cancer cells, whereas apoptosis was induced in Jurkat cells. These findings highlight the biomedical potential of Muricidae extracts in the development of a natural therapy for the treatment of neoplastic tumors and lymphomas.

## 1. Introduction

**Figure 1:**
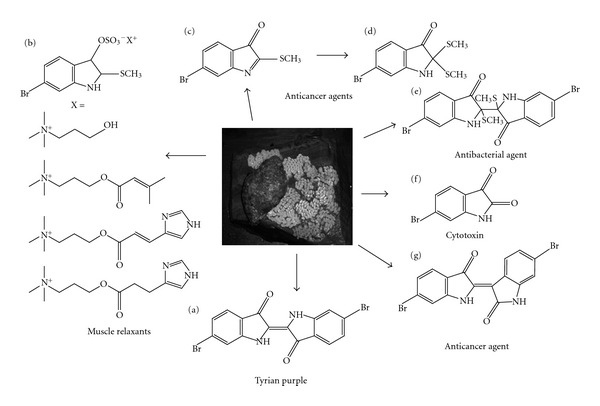
The Australian muricid *Dicathais orbita* pictured with freshly laid egg capsules, which are the source of biologically active secondary metabolites. (a) 6,6′-Dibromoindigotin; (b) tyrindoxyl sulphate salt, where X = choline esters; (c) tyrindoleninone; (d) tyrindoleninone; (e) tyriverdin; (f) 6-bromoisatin and (g) 6,6′-dibromoindirubin.

Many marine secondary metabolites possess biological activities with implications for application as antibiotic, antiparasitic, antiviral and anticancer agents [1–4]. Marine molluscs are the source of at least four structurally distinct anticancer agents, which are currently in phase II and III clinical trials [5, 6]. The Muricidae (Neogastropoda: Mollusca) are a cosmopolitan family of predatory marine gastropods that are historically known for the production of the ancient dye Tyrian purple (Figure 1). The main dye pigment, 6,6′-dibromoindigotin (Figure 1(a)) was the first marine natural product reported in the literature and has attracted substantial scientific interest [7–11]. Tyrian purple is generated from the secretions of the hypobranchial glands of these molluscs, after a series of enzymatic, oxidative and photochemical reactions [9, 11] from a choline ester precursor salt of tyrindoxyl sulphate [9, Figure 1(b)]. Tyrian Purple and its intermediate brominated indole precursors (Figures 1(c)–1(e)) are also found in muricid egg masses [8, 11–13], where they are thought to chemically defend the developing larvae against microbial infection. Preliminary studies on egg mass extracts suggest these intermediate precursors possess anticancer activity [11, 14], in addition to the previously described antibacterial activities [8]. These unique bioactive secondary metabolites are in the chemical class of indole alkaloids from which 60% of all medicines are derived [3].

The brominated intermediate precursor 6-bromo-2-methylthioindolin-3-one (tyrindoleninone, Figure 1(c)) has been identified as a useful anticancer drug lead with apparent specificity toward lymphoma cells (LC_50_ 4 *μ*M) compared with freshly isolated, human mononuclear cells (LC_50_ 195 *μ*M) [14]. However, synthesis of this substance has been problematic due to the instability and rapid oxidation of the precursors and final product. Nevertheless, a number of related substituted isatin derivatives has been synthesized and tested for anticancer activity against a range of human cancer cell lines [14]. The synthesized isatins include 6-bromoisatin (Figure 1(f)), a natural oxidative by-product of Tyrian purple synthesis, which is also found in Muricidae egg masses [8, 12]. Bromination was found to increase the anticancer activity of these isatins and greater selectivity was identified toward leukemia and lymphoma cells over breast, prostate and colorectal carcinoma cell lines [14].

Dye secretion [10] and extracts from the hypobranchial [9] and reproductive glands of male muricids [15] have also been shown to contain the structural isomer of Tyrian purple, 6,6′-dibromoindirubin (Figure 1(g)). Bioassay-guided fractionation of hypobranchial gland secretions have identified this isomer as a potent protein kinase inhibitor [16, 17]. By selectively targeting glycogen synthase kinase-3 (GSK-3), these indirubin compounds effectively inhibit cell proliferation [16, 17]. Indirubin derivatives have also been shown to inhibit Stat3 signaling, inducing apoptosis in human breast and prostate cancer cells [18]. They also suppress tumor necrosis factor (TNF)-induced NF-*κ*B activation in human leukemia and lung adenocarcinoma cells [19] and significantly block proliferation in lung carcinoma, stomach carcinoma and fibrosarcoma cell lines [20]. Furthermore, 5-methyl-indirubin displays anti-tumor activity in lung, renal and prostate *in vivo* models for human cancer [21]. Notably, indirubin has also been identified as the active ingredient in the traditional Chinese herbal medicine Danggui Luhui Wan used to treat leukemia [22].

The homeopathic remedy *Murex* (*purpurea*) (hereinafter referred to as “the *Murex* remedy”) is also derived from dye secretions of the Muricidae and has been in use since the 1800s [23]. The *Murex* remedy is listed on the *Homeopathic Material Medica* for use against a range of “Women's problems” including uterine and breast cancer [24, 25]. In most cases there is little scientific data available to support the biological activity of homeopathic remedies and few have been tested for safety and effectiveness using rigorous methodologies [26, 27]. Nevertheless, some homeopathic drugs have recently been tested and shown to have anti-cancer effects (e.g., [28] and references therein). The apparent biological activity of organic extracts derived from the Muricidae (see [8, 11, 16, 29]; Figure 1) suggests that there may also be some chemical basis to the homeopathic application of the *Murex* remedy.

Here we investigate the compounds present in the *Murex* remedy and compare them to brominated secondary metabolites identified in crude extracts obtained from the egg masses of *Dicathais orbita*, a common species of Muricidae located along the temperate coasts of Australia and New Zealand [30]. A semi-purified extract was also prepared from the crude egg mass to concentrate Tyrian purple precursors, in particular tyrindoleninone (Figure 1(c)). The anti-proliferative and cytotoxic effects of crude and semi-purified egg extracts, as well as the *Murex* remedy were compared on a range of human carcinoma, lymphoma and normal mononuclear cells.

## 2. Methods

### 2.1. Collection of Egg Masses and Preparation of Solvent Extracts

The egg masses of *D. orbita* were collected from coastal intertidal reefs along the Fleurieau Peninsula, South Australia and from a breeding population held in a recirculating marine aquarium system at Flinders University. A total of 70 g of egg capsules were collected, wrapped in aluminum foil and stored at −70°C.

Egg capsules were sliced open in minimum light and during the extraction procedure, all glassware was covered in alfoil to exclude light. Organic extracts were prepared by soaking in 100 mL (per 10 g eggs) AR grade chloroform and methanol (1 : 1, v/v, Selby-Biolab, Pronalys, Melbourne, Australia) under agitation at 4°C for 2 h, followed by overnight soaking in fresh solvent then a final 2 h extraction. The extracts were then combined, filtered and distilled water (50 mL) added, to enable separation of the chloroform layer, which was then dried in a rotary evaporator under reduced pressure with water pump vacuum (337 Hg) at 40°C. This extraction procedure enables concentration of the bioactive indole precursor, tyrindoleninone, in the chloroform layer [8], and although some tyrindoxyl sulphate salt may be discarded in the polar layer, preliminary bioassays indicate the polar extract has relatively limited anticancer activity (unpublished data). Extracts were transferred to amber vials in approximately 1 mL of 100% DCM, dried under N_2_ gas and stored in amber vials at −20°C to prevent degradation.

In order to concentrate tyrindoleninone, the crude egg extracts (1 g) were semi-purified using flash silica chromatography pressurized with nitrogen gas, as previously described [8]. The extract was passed through a silica column (40 g, 230–400 silica mesh, Merck) using high performance-liquid chromatography (HPLC) grade dichloromethane (DCM, Sigma-Aldrich, Chromasolv, Sydney, Australia) as the elution solvent. Fractions were collected from the column by visual monitoring of the colored compounds, with the bright orange fraction containing the previously identified tyrindoleninone [8, 31]. Semi-purified fractions were dried and stored as outlined above.

### 2.2. The Murex Remedy

The homeopathic remedy *Murex Purpurea* (1 M) was purchased from Washington Homeopathic Products (Berkeley Springs, WV, USA). This diluted tincture is supplied in 20% EtOH and water. To concentrate the remedy, 60 mL was dried in a rotary evaporator with pump vacuum (72 Hg) at 40°C. The evaporated remedy was then dissolved in DCM, transferred to an amber vial and stored at −20°C.

### 2.3. Chemical Analysis

All extracts and the concentrated *Murex* remedy were analyzed by HPLC (Waters Alliance) coupled to a mass spectrometer (MS, Micromass Quatro micro) according to Westley and Benkendorff [15]. Parallel UV/Vis diode-array detection (DAD) was used at 300 nm and 600 nm and compounds entered into the mass spectrometer with electrospray ionization (ESI). Major ions in ESI-MS were obtained at the apex of HPLC peaks and compounds were identified by the retention time and characteristic mass ion clusters for Br^79^ Br^81^ [15]. Relative proportions of each compound were calculated from integrated absorption data at 300 nm using MassLynx 4.0 software.

### 2.4. Cell Culture

Five human cancer cell lines (Caco-2, colon carcinoma; HT29, colon carcinoma; MCF-7, breast carcinoma; Jurkat, T cell lymphoma and U937, histiocytic lymphoma) and an untransformed rat small intestine epithelial cell line (IEC-6) were maintained at 37°C in a 5% CO_2_ humidified atmosphere. HT29, MCF-7, Jurkat and U937 cells were maintained in RPMI-1640 medium (Sigma) supplemented with 10% fetal bovine serum (FBS, JRC Biosciences, Lenexa, KS, USA), Penicillin/Streptomycin (Gibco, Invitrogen Corporation, Bethesda, MD, USA) and 200 nM L-glutamine (Sigma). Caco-2 and IEC-6 cells were maintained in Dulbecco's Modified Eagle's Medium **(**DMEM, Sigma) supplemented with 20% and 10% FBS, respectively, Penicillin/Streptomycin (Gibco) and 200 nM L-glutamine (Sigma). Human mononuclear cells (MNC) were harvested from two healthy donors. Cells were isolated using a separation gradient (Lymphoprep, Nycomed Pharma AS, Oslo, Norway) and 1 × 10^6^ MNC were suspended in RPMI-1640 supplemented medium and 100 *μ*L/well placed in 96-well plates. MNC were treated on the same day as harvesting.

### 2.5. Cell Proliferation Assay

Adherent cells (HT29, Caco-2, MCF-7, HeLa and IEC-6) were grown to 70% confluence then detached from flasks with 1 × Trypsin-EDTA (Gibco) and resuspended at 1 × 10^5^ cells/mL. Cells were dispensed into 96-well plates (Nunc, Roskilde, Denmark) at a final concentration of 1 × 10^4^ cells/well and incubated overnight. Media was removed and replaced prior to treatment. Suspension cell lines (Jurkat and U937) and MNC were washed once with PBS and suspended at 1 × 10^5^ cells/mL. Cells were dispensed at a final concentration of 1 × 10^4^ cells/well and incubated overnight before treatment. The effect of the test samples on cell viability was measured using the MTS cell proliferation assay, which measures the reduction of MTS tetrazolium salt to formazan in metabolically active cells [32].

Due to the poor solubility of crude and semi-purified extracts in aqueous solution, extracts were dissolved in 100% dimethylsulphoxide (DMSO, Sigma, USA) fresh on the day of testing and added to the cell cultures (in triplicate), ranging in concentration from 0.01 mg/mL to 1 mg/mL (final DMSO concentration of 2%). The concentrated *Murex* remedy was also dissolved in DMSO and tested at a final concentration 4 mg/mL. The diluted *Murex* remedy was tested neat by adding 2 *μ*L to 98 *μ*L of cell culture (i.e., 50-fold dilution). According to the manufacturers recommendations the *Murex* remedy (1 M) should be diluted before use (four drops into one teaspoonful H_2_O), therefore our tests of the diluted product provide a relevant test of bioactivity for homeopathic application. Appropriate media and solvent (2% DMSO for the concentrated extract and 20% EtOH in water for the dilute *Murex*) controls were also prepared in triplicate. All samples were incubated with the cells for 4 h before 20 *μ*L of MTS [3-(4,5-dimthylthiazol-2-yl)-5-(3-carboymethoxyphenyl)-2-(4-sulfophenyl)-2H-tetrazolium, inner salt] reagent (Promega Co. Madison, WI, USA) was added to each well. Cells were incubated for a further 1.5 h to allow for color development before the absorbance values were read at 492 nm using a Multiscan plate reader (Labsystems, Virginia, USA) and Accent software 2.6. Background absorbance from controls involving the test samples incubated in media with the MTS reagent, were subtracted from sample wells after the final absorbance was obtained. Repeat assays were conducted on all samples on at least three separate occasions with independent extracts, with the exception of the concentrated and dilute *Murex* remedy which was tested in quadruplicate and repeated on two separate occasions.

### 2.6. Statistical Analysis

All treatment (crude extract, semi-purified and the *Murex* remedy) data points were normalized to the relevant solvent controls and expressed as percentage cell viability. The data are represented as the average of triplicate samples and are expressed as the mean ± SE of repeat experiments. Significant differences in percentage viability between the different treatments concentrations were tested using one way Analysis of Variance (ANOVA), or the Kruskal-Wallis test for nonparametric data, on SPSS 14.0 statistical software. *P* < .05 was used to establish significant dose effects for each test sample against each cell line.

### 2.7. Flow Cytometry Analysis

HT29 and Jurkat cells were cultured in 24-well plates (in duplicate) with 1 × 10^5^ cells/well, incubated overnight and treated with extracts the following day as described above. Adherent cells were detached from the wells using 1x trypsin-EDTA solution (Gibco), and manual pipetting. Cells were placed in 10 mL tubes before centrifugation at 150 rpm for 3 min. Media was removed without disturbing the cells. The pellet was washed twice with cold sterilized phosphate buffered saline (PBS, pH 7.4) and centrifuged. Cells were then suspended in 200 *μ*L of 1x Binding buffer (10 mM Hepes/NaOH, pH 7.4, 140 mM NaCl and 2.5 mM CaCl_2_). Prior to analysis, propidium iodide (PI, Sigma) at 10 *μ* g/mL final concentration and 5 *μ*L Annexin-V-FITC (BD Biosciences, NJ, USA) were added to each tube. Analyses were performed using FACSan Flow Cytometer (Becton Dickinson) with the CellQuest program (Becton Dickinson) and FlowJo 7.2.2 analysis software (Tree Star Inc.). Compensation was performed with appropriate solvent controls for each analysis.

## 3. Results

### 3.1. Chemical Analysis

**Figure 2:**
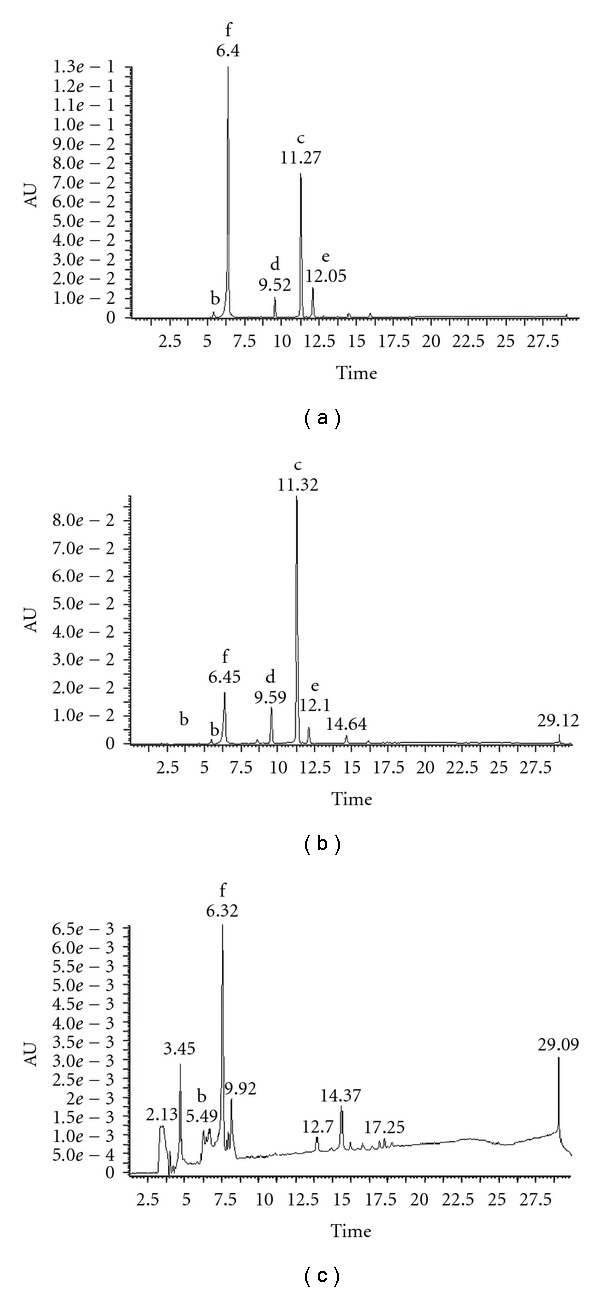
LC-MS analysis showing representative chromatograms from the UV diode array at 300 nm; (a) Crude extract from egg capsules of *D. orbita*; (b) Semi-purified fraction from the crude extract and (c) the *Murex* remedy. The peaks correspond to brominated indoles, where b, tyrindoxyl sulphate; c: tyrindoleninone; d: tyrindolinone; e: tyriverdin; f: 6-bromoisatin. Note that due to the low detection of any compounds in the concentrated *Murex* remedy sample, the *y*-axis has an intensity of approximately 10 times less than the egg extracts.

LC-MS analysis of chloroform-soluble extracts from *D. orbita* egg masses revealed five peaks corresponding to brominated indoles (Figure 2). The dominant compound present in the crude extract (Figure 2(a)) registered an HPLC peak at 6.4 min and major ions at *m/z* 224, 226, that can be attributed to the pseuomolecular ion [M-H]^+^ of 6-bromoisatin (Figure 1(f), molecular mass of 225, 227 for Br^79^, Br^81^). Another dominant peak occurred at 11.3 min with major ions in ESI-MS at *m/z* 255, 257 corresponding to the molecular mass of tyrindoleninone (Figure 1(c)) and a smaller peak at 9.5 min had *m/z* 303, 305 corresponding to the molecular weight of tyrindolinone [6-bromo-2,2-dimethylthioindolin-3-one] (Figure 1(d)) a methane thiol adduct of tyrindoleninone. An additional peak at 12.1 min registered an isotopic cluster at *m/z* 513, 515, 517 (in a peak height ratio of 1 : 2 : 1) corresponding to the molecular ion of tyriverdin (MH^+^; Br^79^ Br^79^, Br^79^ Br^81^, Br^81^ Br^81^). Additional fragment ion triplets (indicating two bromines still attached) were centered around *m/z* 465 (Br^79^ Br^81^) from the elimination of a single methane thiol group, and majors peaks at *m/z* 419 [Br^79^ Br^81^ (MH-2SCH_3_)^+^] confirming this peak as tyriverdin (Figure 1(e)). A minor HPLC peak at 5.1 min in the crude extract (Figure 2(a)) corresponds to tyrindoxyl sulphate (Figure 1(b)), with major ions in ESI-MS at *m/z* 338, 336.

LC-MS analysis revealed that semi-purification of the *D. orbita* egg extract (Figure 2(b)) by silica chromatography was successful in concentrating tyrindoleninone (c). The relative proportion of tyrindoleninone increased from 31.12% of the crude extract (Figure 2(a)) to 61.72% of the semi-purified fraction (Figure 2(b)) based on spectral analysis of absorption at 300 nm. There was a corresponding decrease in the relative proportion of 6-bromoisatin (f) from 52.97% of the crude extract (Figure 2(a)) to 18.88% in the semi-pure fraction (Figure 2(b)). Tyrindolinone (d) and tyriverdin (e) remained present as minor components in the semi-pure fraction (Figure 2(b)).

It was difficult to register any compounds from peaks absorbing in the UV-Vis range from the diode array chromatogram of the concentrated *Murex* remedy. However, after decreasing the *y*-axis scale to intensify the peaks, two brominated compounds were detected at 300 nm (Figure 2(c)). At 6.3 min, 6-bromoisatin (f, M^+^ 225, 227) was detected representing a relative proportion (59.35%) of the integrated area from all peaks registering at 300 nm. A co-eluting peak at 5.4 min was identified as tyrindoxyl sulphate (b, M^+^ = 338, 336), representing approximately 9.1% of the concentrated *Murex* remedy extract. None of the other peaks produced isotopic ion clusters characteristic of brominated compounds or molecular ions corresponding to indoles as previously recorded in Muricidae extracts [9, 12, 15].

### 3.2. Effects on Cell Viability

**Figure 3:**
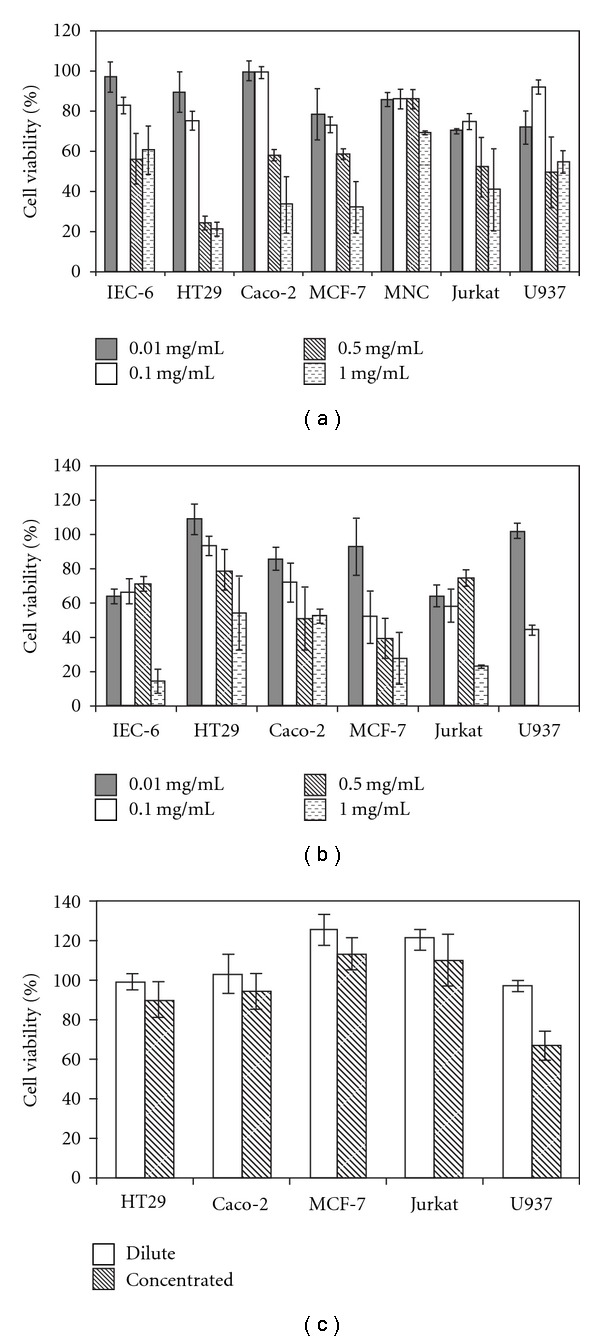
Percent cell viability of a range of solid and haemopoietic cell lines exposed for 4 h to varying concentrations of (a) crude extract; (b) semi-purified fraction (concentrating the tyrindoleninone compound) from *D. orbita* and (c) the *Murex* remedy. Cell viability is determined by the percent absorbance at 490 nm relative to solvent controls, based on the conversion of a tetrazolium salt to formazan in the MTS cell proliferation assay. Cells were treated in triplicate and the assay repeated on separate occasions, shown as mean ± SE of repeat experiments.

The DMSO solutions of the chloroform-soluble crude extract from *D. orbita* egg masses were found to reduce the production of formazan relative to solvent controls in all cell lines at a concentration of 1 mg/mL, with additional effects at lower concentrations with all solid tumor and U937 lymphoma cells (Figure 3(a)). At the highest concentration of 1 mg/mL, a mean reduction of 39.7% cell viability was observed for the untransformed IEC-6 cells (Figure 3(a)), compared with a 78.5% mean reduction in HT29 colorectal carcinoma and 66.7% mean reduction in Caco-2 colorectal carcinoma cell viability. Reductions in cell viability also occurred in these solid tumors incubated with 0.5 mg/mL crude extract, which caused over 70% reduction in formazan production for HT29 cells and over 40% reduction for Caco-2 and MCF-7 cells. Further reductions of over 20% cell viability were observed at 0.01 mg/mL for HT29 cells, as well as in MCF-7 cells incubated with both 0.1 mg/mL and 0.01 mg/mL extract. Significant dose effects were observed in all solid tumor cell lines (*P* < .05), with lower cell viability recorded at the higher treatment concentrations (Figure 3).

Semi-purification of the *D. orbita* egg chloroform-soluble extract was successful in increasing the effects on cell viability against some of the adherent cell lines (Figure 3), but in a non-predictable manner. Stronger activity was observed against the untransformed IEC-6 rat epithelial cells (Figure 3(b)), with significantly lower cell viability at 1 mg/mL compared with the other test concentrations. Greater activity of the semi-purified extract was also observed against Caco-2 cells at the lower test concentrations, with over 60% reduction in cell viability at 0.1 mg/mL (Figure 3(b)). In comparison to the crude extracts (Figure 3(a)), MCF-7 breast carcinoma cells also showed greater mean reduction in cell viability (>50%) when incubated with 0.1–1 mg/mL of the semi-purified extract (Figure 3(b)), However, less activity was observed against the colorectal carcinoma cell line HT29 (Figure 3(b)). No significant dose effects were observed with the semi-purified extracts against any of these solid tumors (*P* > .05).

Treatment of the T cell lymphomas with the crude extracts resulted in reductions of over 50% cell viability at 1 and 0.5 mg/mL (Figure 3(a)). By comparison, treatment of normal MNC resulted in only a small reduction in cell viability at the highest concentration (1 mg/mL, 30.8% reduction). Unlike the adherent cell lines, no significant dose effects were observed for the Jurkat and U937 cells, but rather, relatively large variation was observed between repeat assays and replicates (Figure 3(a)). Much stronger effects on cell viability were found with the semi-purified extracts against both the lymphoma cell lines (Figure 3). For the Jurkat cells, there was a significant reduction in cell proliferation by almost 80% when treated with the semi-purified extracts at 1 mg/mL. Further effects of the semi-purified extracts were observed at the lower concentrations of 0.01 and 0.1 mg/mL, with less than 80% of the cells remaining viable (Figure 3(b)). In U937 cells, a reduction in cell viability to around 50% of the solvent control resulted after incubation with the semi-purified extract at 0.1 mg/mL, compared with over 90% cell viability with the crude extract at this concentration (Figure 3). Cell viability of U937 cells was significantly reduced by treatment with the semi-purified extract at 0.1 mg/mL compared with 0.01 mg/mL (*P* = .0001).

No reduction in cell viability was seen in the majority of cell lines treated with the dilute *Murex* remedy (Figure 3(c)). Similarly, no effects were seen with the concentrated *Murex* remedy (4 mg/mL), with the exception of the histiocytic lymphoma cell line U937, in which cell viability was reduced by 35% (Figure 3(c)). Cell viability of U937 cells was significantly lower after treatment with the concentrated product compared with the dilute remedy (*P* = .004). A mild increase in the amount of formazan production was observed in HT29, Caco-2 and MCF-7 cells treated with the dilute *Murex* remedy relative to solvent controls (mean > 100%, Figure 3(c)), although no statistically significant difference in cell viability was detected relative to the concentrated treatments.

### 3.3. Initiation of Necrosis and Apoptosis

**Figure 4:**
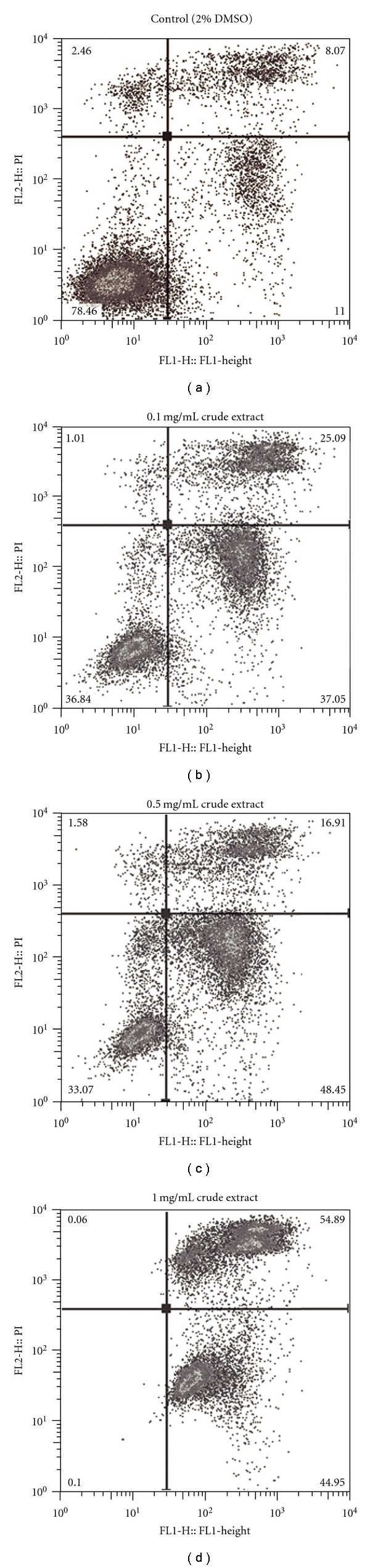
Representative flow cytometric analysis of Jurkat T cell lymphoma cells (1 × 10^5^) treated with; (a) DMSO only (final concentration 2%); (b) 0.1 mg/mL of crude extract; (c) 0.5 mg/mL of crude extract and (d) 1 mg/mL crude extract from *D. orbita* for 4 h. FL1 (*x*-axis) shows Annexin-V positive cells, FL2 (*y*-axis) shows propidium iodide (PI) positive cells. Cells were treated in duplicate, dual stained with PI and Annexin-V-FITC and analyzed using a FACscan flow cytometer and FlowJo analysis software.

**Figure 5:**
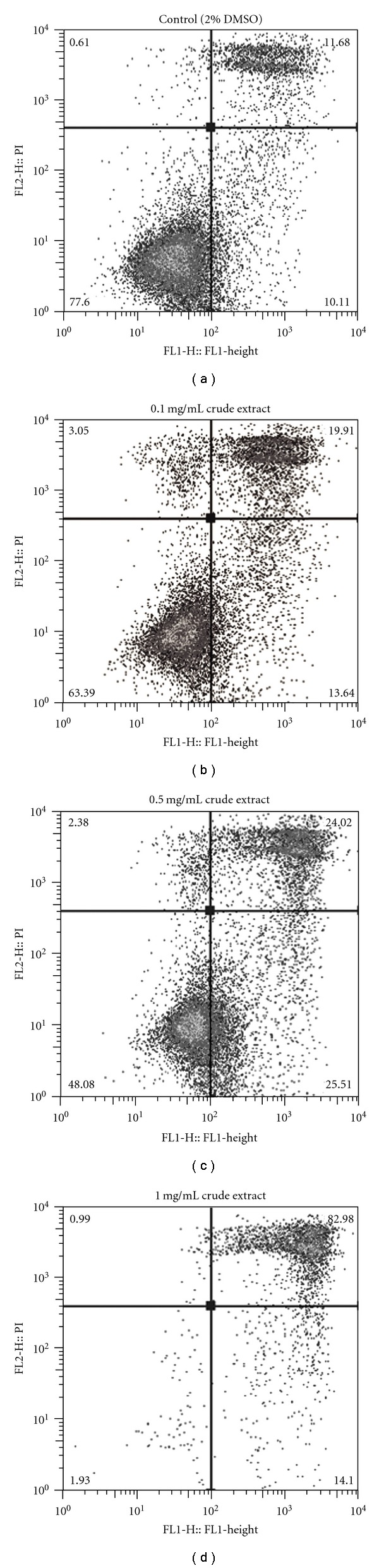
Representative flow cytometric analysis of HT29 colorectal carcinoma cells (1 × 10^5^) treated with; (a) DMSO only (final concentration 2%); (b) 0.1 mg/mL of crude extract; (c) 0.5 mg/mL of crude extract and (d) 1 mg/mL crude extract from *D. orbita* for 4 h. FL1 (*x*-axis) shows Annexin-V positive cells, FL2 (*y*-axis) shows propidium iodide (PI) positive cells. Cells were treated in duplicate, dual stained with PI and Annexin-V-FITC and analyzed using a FACscan flow cytometer and FlowJo analysis software.

**Figure 6:**
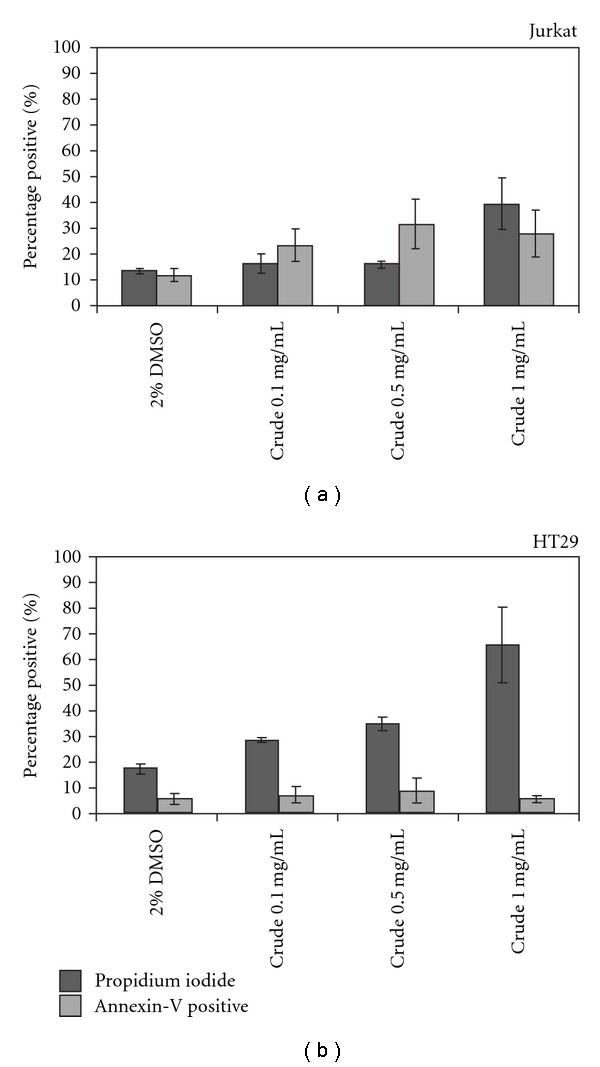
Mean (±SE) proportion of gated cell populations from repeat flow cytometry experiments for (a) Jurkat (T cell lymphoma) cells and (b) HT29 (colorectal carcinoma) cells demonstrating the percentage of cells positive for PI and Annexin-V.

Flow cytometric analysis revealed that Jurkat cells treated with 1 mg/mL DMSO solution of chloroform-soluble crude extract for 4 h (Figure 4(d)) underwent an induction of both necrosis (54.89% PI positive) and apoptosis (44.95% Annexin-V positive) compared with the treatment control (11% Annexin-V positive, 8.07% PI positive, Figure 4(a)). In contrast, HT29 cells treated with the DMSO solution of chloroform-soluble crude extract were predominantly PI positive (Figures 5(b)–5(d)), indicating that they were undergoing necrosis. At 1 mg/mL, over 80% of HT29 cells were PI positive (Figure 5(d)), compared with 11.68% of the solvent control cells (Figure 5(a)). An increase in the proportion of PI positive cells was also observed in HT29 cells treated with 0.5 (Figure 5(c)) and 0.1 mg/mL (Figure 5(b)) compared with the solvent control (Figures 5(a) and 6(b)), whereas an increase in PI positive Jurkat cells was only observed at the highest concentration of 1 mg/mL (Figure 6(a)). On average, almost double the amount of Jurkat cells were Annexin-V positive at the lower concentration of 0.5 mg/mL, when compared with the solvent control (Figure 6(a)), whereas no obvious increases the proportion of Annexin-V positive cells was observed for HT29 cells treated at any concentration of the egg extract (Figure 6(b)).

## 4. Discussion

This study confirms the presence of brominated indoles with anticancer activity in extracts from the egg masses of the Australian mollusc *D. orbita*. Despite the detection of trace amounts of two brominated indoles in the commercially available *Murex* remedy, no significant effects on cell viability were observed with extracts of this commercial product in a wide range of cell lines, with the exception of a mild reduction in the histiocytic lymphoma cell line U937 after treatment with the concentrated product. Consequently, the anticancer applications of this natural remedy cannot be substantiated by our study. Nevertheless, the broad spectrum reduction in cell viability in solid tumor and lymphoma cell lines treated with our crude and semi-purified egg extracts imply that there is potential for optimizing a natural anticancer remedy from the Muricidae. The crude extract was found to have differential effects depending on cell phenotype, inducing both apoptosis and necrosis in Jurkat cell lines, whereas strongly necrotic effects were observed with HT29 carcinoma cells.

Anti-proliferative effects in the crude extract obtained from the egg masses of *D. orbita* were predominantly seen in the cancer derived cell lines, with less activity towards the untransformed rat small intestine epithelial cell line IEC-6 and the freshly isolated MNC. This dose dependant specificity towards transformed cancer cell lines is useful because non-specific cytotoxicity towards healthy cells is a major limitation in the development of many marine natural products as anticancer agents [4, 33]. Semi-purification of the egg extract was successful in increasing the relative concentration of tyrindoleninone relative to that of 6-bromoisatin. However, this resulted in increased activity towards benign gut epithelial cells and did not consistently increase the potency towards all the solid tumor cell lines tested. This indicates that some cell lines (e.g., HT29) are relatively resilient to the anti-proliferation effects of tyrindoleninone, compared with the known cytotoxic effects of 6-bromoisatin [11, 14], whereas other cell lines (e.g., IEC-6 and the lymphoma cells) are more susceptible to higher concentrations of tyrindoleninone. These observed differences in cell line specificity may indicate the bioactive compounds within the extracts target alternative pathways depending on cell phenotype, as suggested by Nguyen and Wells [34].

Our results indicate that there is potential for optimizing the composition and delivery of Muricidae extracts for the treatment of different cancers by concentrating the different brominated indoles. The strongest effects on cell viability using the crude *D. orbita* extract were exerted against the HT29 colon carcinoma cells. This is in contrast to previous studies on the anticancer effects of indole derivatives, where Vine et al. [14] reported greater effects on lymphoma cells compared with solid tumors in a range of synthetic isatin derivatives and Hoessel et al. [22] found HT29 cells to be insensitive to indirubin-3′-monoxime. The relatively strong selective inhibition observed towards colon carcinomas treated with the *D. orbita* crude extract is of particular interest considering that *D. orbita* is an edible species of marine mollusc. The bioactive brominated indoles found in the chloroform-soluble egg extracts are also present in the hypobranchial and reproductive organs of adult *D. orbita* [15] and appear to be retained after boiling the snails (KB, unpublished data). The Muricidae are heavily fished as a source of protein in many countries around the world [35] suggesting potential for development as a medicinal food. In some cultures (e.g., Asia), Muricidae are often consumed whole, with the hypobranchial gland intact (KB, personal observation), whereas in other cases (e.g., Mediterranean) the hypobranchial gland is typically discarded along with the visceral organs. Our tasting of the hypobranchial glands from *D. orbita* indicates that it is bitter and leaves a numbing effect on the mouth, most likely due to the muscle relaxing choline esters which are also produced in this organ [10, 36]. Further studies are required to establish whether the bioactive compounds from Muricidae are nontoxic and bioavailable after digestion.

In contrast to the HT29 carcinomas, greater activity was observed against the U937 and Jurkat lymphoma cells using the semi-purified extract. Nevertheless, the level of activity observed was somewhat disappointing based on a previous study [11], where *D. orbita* egg extracts were reported to cause over 80% reduction in the cell viability of U937 cells after 1 h incubation at 0.01 mg/mL. Furthermore, the level of activity observed in the semi-purified extract is nowhere in the order of the LC_50_ of 4 *μ*M predicted by Vine et al. [14] for tyrindoleninone. These differences could be due to variability in growth parameters of different cultures of the same cell line [37, 38] or variability in proportion of bioactive compounds present in the extracts from the egg masses. It is also possible that the contaminating plasticizer in the previous studies [14] acted synergistically with tyrindoleninone by increasing its solubility and or stability in the media. This would increase its bioavailability to the cells at low concentrations, in comparison to the strongly lipophylic nature of our extracts, which could lead to micelle formation in the aqueous media. Attempts by Vine et al. [14] have failed to synthesize tyrindoleninone in the laboratory, due to instability of the precursors with O_2_ exposure. Consequently, it will be difficult to establish the true effective lethal dose or minimum inhibitory concentration of this biologically active marine natural product.

**Figure 7:**
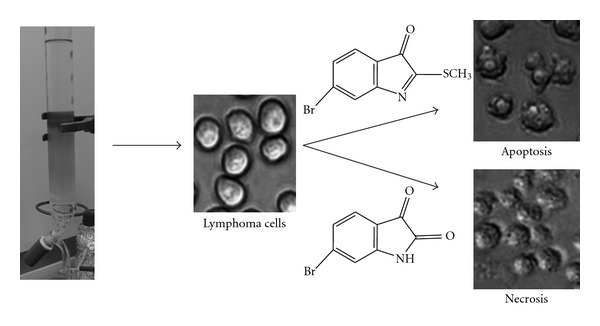
Chloroform extracts from *D. orbita* egg masses contain at least two brominated indoles with anti-cancer activity that can be purified by silica chromotography. The orange fraction contains tyrindoleninone, which is predicted to induce apoptosis in lymphoma cells, whereas the more polar yellow compound is the cytotoxin 6-bromoisatin, which causes necrosis in lymphoma and solid tumor cell lines.

The differential activity of *D. orbita* extracts towards solid tumors and suspension cells could have useful implications for understanding the mode of action and in particular, whether the bioactive compounds effectively induce programmed cell death. Nguyen and Wells [34] found that apoptosis was not induced in all cell lines while investigating compound effects on cytochrome *c*-induced caspase activation and attributed this to the possibility that differential expression of various pro- and anti-apoptotic factors may provide a strategy for selectivity. An interesting finding from our flow cytometry analyses was the increase in apoptosis in Jurkat cells treated with all concentrations of the extracts relative to the solvent controls. However, HT29 treated cells predominantly underwent necrosis with little increase in Annexin-V positivity. This is consistent with previous reports that apoptosis is characteristic for lymphoid cells, but is an insignificant mode of cell death for many solid tumors [39]. The necrotic effects are most likely attributed to 6-bromoisatin, the dominant compound in the chloroform-soluble crude extract (Figure 7), as previous studies have indicated the cytotoxic behavior of brominated isatin derivatives at concentrations greater than 10 *μ*g/mL [14]. It is unclear why Jurkat cells appear to be less susceptible to these cytotoxic effects, although semi-purification of the extracts to reduce bromoisatin relative to tyrindoleninone could reduce the necrotic effects on both cells lines (Figure 7). Further investigation into the mode of action of our semi-purified extracts and/or the isolated bioactive secondary metabolites is warranted, to determine which cellular pathways are targeted by these compounds.

Using LC-MS analysis we identified the presence of 6-bromoisatin and trace amounts of tyrindoxyl sulphate in the *Murex* remedy. The presence of tyrindoxyl sulphate confirms the *Murex* remedy as originating from the hypobranchial glands of Muricidae, as this compound was first reported from *D. orbita* [40] and has only subsequently been reported from other species in the same family of molluscs [9]. However, despite the fact that this secretion is sourced from the “purple secretion”, the Tyrian Purple pigments 6,6′-dibromoindigotin and its biologically active structural isomer 6,6′-dibromoindirubin, were not detected. This is possibly due to the insolubility of these hydrophobic compounds in 20% aqueous ethanol, which is the delivery agent used in the homeopathic product. The presence of 6-bromoisatin implies that at least some tyrindoxyl sulphate has undergone enzymatic cleavage or acid hydrolysis and oxidation to enable formation of the end products [9]. But despite the presence of 6-bromoisatin, we observed no significant reduction of cell viability in any of the solid tumors and only a slight reduction in one lymphoma cell line tested using the concentrated *Murex* remedy. Consequently, 6-bromoisatin must not be present in sufficient quantities in the *Murex* remedy to exert a strong effect, possibly due to dilution with other compounds that could not be detected in the LC-MS. Previous chemical studies on homeopathic solutions have indicated that the results often cannot be reproduced [41]. Similarly, our LC-MS on different batches of the *Murex* remedy indicated variability in the detection of 6-bromoisatin (data not shown), although all of our assays were performed on a batch containing the maximum concentration of this compound as shown in Figure 2(c). Homeopathic practitioners frequently prescribe medicines in extremely high dilutions, including dilutions whereby not a single molecule of the starting substance is present (“ultramolecular” dilutions) [42]. Therefore, in many cases anecdotal reports of the benefits of homeopathic treatment may be attributed to the placebo effect. Homeopathy is drawn from the central principle of treating like with like [42] and notably, there was a slight (non-significant) increase in the cell proliferation of solid tumors treated with the dilute remedy. Nevertheless, these effects were not increased in the concentrated product, as would be expected for a mild promoter of cell replication. Consequently, our results suggest that the use of the *Murex* remedy remains unsubstantiated as a natural homeopathic remedy for the treatment of cancer. Given that the main application of the *Murex* remedy is for Women's problems, our ongoing research will continue to explore the effects on cell proliferation and hormone production in uterine cancer and granulosa cells.

Here we have identified bioactive brominated indoles in the commercially available *Murex* remedy and extracts from the fresh egg capsules of the Australian muricid *D. orbita*. Concentration of the *Murex* remedy did not significantly reduce cell viability in a metabolic cell proliferation assay, in most of the cell lines tested. In contrast, both crude and semi-purified extracts significantly reduced proliferation in a range of solid tumor and lymphoma cell lines. The strongest anti-proliferative activity in the crude extract was observed against HT29 colon carcinomas and flow cytometry revealed significantly increased necrosis compared with treatment controls after only 4 h. The crude extract was found to induce apoptosis and necrosis in Jurkat T cell lymphoma cells (Figure 7) and semi-purification of the extract to concentrate tyrindoleninone resulted in increased effects on the cell viability of both lymphoma cell lines tested in the MTS assay. These results indicate the potential to optimize bioactive muricid extracts for development as alternative medicines for the treatment of cancer.
